# DCD – a novel plant specific domain in proteins involved in development and programmed cell death

**DOI:** 10.1186/1471-2105-6-169

**Published:** 2005-07-11

**Authors:** Raimund Tenhaken, Tobias Doerks, Peer Bork

**Affiliations:** 1 Plant Molecular Biology, University of Frankfurt, Marie-Curie-Str. 9, 60439 Frankfurt, Germany; 2 European Molecular Biology Laboratory, Meyerhofstr. 1, 69102 Heidelberg, Germany

## Abstract

**Background:**

Recognition of microbial pathogens by plants triggers the *hypersensitive reaction*, a common form of programmed cell death in plants. These dying cells generate signals that activate the plant immune system and alarm the neighboring cells as well as the whole plant to activate defense responses to limit the spread of the pathogen. The molecular mechanisms behind the *hypersensitive reaction *are largely unknown except for the recognition process of pathogens. We delineate the NRP-gene in soybean, which is specifically induced during this programmed cell death and contains a novel protein domain, which is commonly found in different plant proteins.

**Results:**

The sequence analysis of the protein, encoded by the NRP-gene from soybean, led to the identification of a novel domain, which we named DCD, because it is found in plant proteins involved in **d**evelopment and **c**ell **d**eath. The domain is shared by several proteins in the *Arabidopsis *and the rice genomes, which otherwise show a different protein architecture. Biological studies indicate a role of these proteins in phytohormone response, embryo development and programmed cell by pathogens or ozone.

**Conclusion:**

It is tempting to speculate, that the DCD domain mediates signaling in plant development and programmed cell death and could thus be used to identify interacting proteins to gain further molecular insights into these processes.

## Background

Plants can recognize microbial pathogens by a specific interaction system, which was historically named the gene-for-gene interaction, because particular matching genes must be present in the pathogen as well as in the plant. A successful recognition triggers a hypersensitive reaction of individual plant cells, which is a form of programmed cell death in plants. Though a dead cell on its own might already stop the growth of biotrophic pathogens more importantly the cell death program by itself generates unknown signals for neighboring cells. Thereby the plant immune system is activated locally in some cell layer around the original infection to prepare the plant cells for the next microbial attack. Often this signal from the first infection spreads throughout the whole plant and turns on a long lasting broad pathogen resistance called the systemic acquired resistance. Despite the enormous efforts to dissect the machinery for the hypersensitive reaction many details are still unknown except for the early recognition of the microbial molecules.

Often the programmed cell death in plants requires the signaling compound salicylic acid downstream of the recognition process to proceed beyond restrictions points in the cell death program [1]. A conclusive role for salicylic acid has not been worked out but it is likely to function in signal amplification [2,3] and transcriptional activation of genes are very likely [4,5].

We have isolated a gene from soybean which is strongly induced during the hypersensitive reaction and serves as a marker for programmed cell death in this system [6]. The gene is not directly responsive to salicylic acid but transcription can be amplified in the presence of this signal molecule. The gene encodes a protein consisting of two domains. The N-terminal domain is extremely rich in the amino acid asparagine (~25%) and was therefore called N-rich protein (NRP) [6]. The exact biological function of the NRP-gene remains to be elucidated.

Here we describe the analysis of a protein domain found in the soybean NRP-protein and other plant proteins associated with development. The biological processes associated with these proteins lead us to name this novel domain DCD for their role in development and cell death.

## Results and discussion

**Figure 1:**
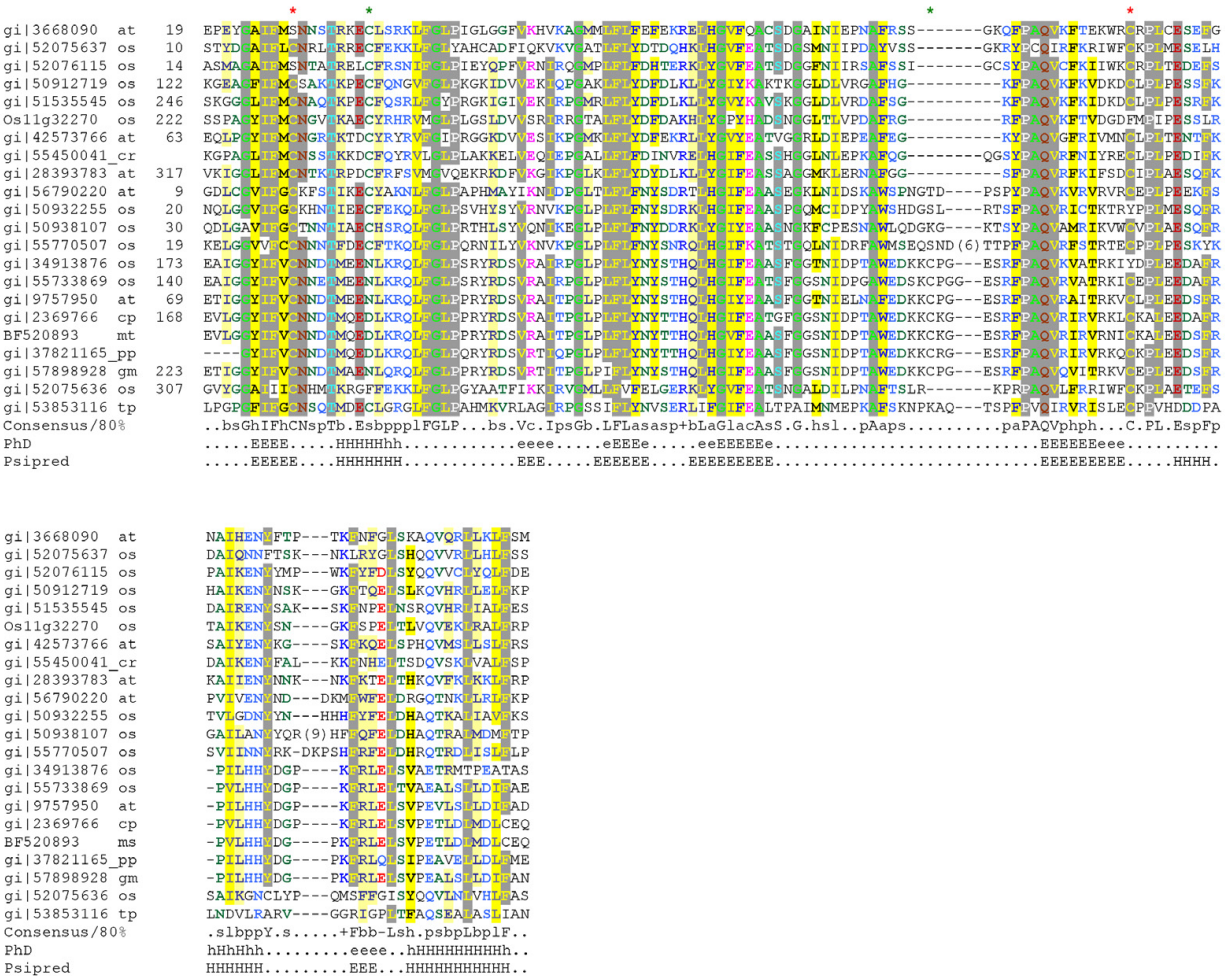
**Multiple sequence alignment of DCD-domains. **The alignment was built using T-coffee [21] and refined manually. First column: database accession numbers (Genbank, if available); second column: species names (at: *Arabidopsis thaliana*; cp: *Citrus X paradisi*; cr: *Ceratopteris richardii*; gm: *Glycine max*; mt: *Medicago truncatula*; os: *Oryza sativa*; tp: *Thalassiosira pseudonana*); third column: start of the domain in the respective sequences. The aligment is coloured by chroma [22]. (conserved prolines: white on grey; conserved glycines and alanines: green on grey; conserved leucines, isoleucines, phenylalanines, cysteines, valines and tyrosines: yellow on grey; conserved asparagines and glutamines: dark red on grey; conserved glutamic acids: light red on grey; conserved threonines and serines: light blue on grey; conserved aliphatic residues: grey on yellow; conserved hydrophobic residues: black on yellow; conserved small residues: dark green on white; conserved positively charged residues: blue on white; conserved polar residues: dark blue on white; conserved charged residues: pink on white; conserved aromatic residues: blue on yellow; conserved big residues: blue on light yellow; conserved negatively charged residues: red on white) The consensus sequence (conserved in 80% of the sequences) shown below; h, p, s, l, b, c, a, + and – indicate hydrophobic, polar, small, aliphatic, big, charged, aromatic, positively charged and negatively charged residues, respectively. The predicted secondary structure taken from the consensus of the alignment (H, helix or E, beta sheet predicted with expected average accuracy > 82%; h, helix or e, beta sheet predicted with expected average accuracy < 82%) using PhD [23]. Independent predictions have been performed for Psipred 17 using representatives of distinct groups (accession number: gi|2369766, gi|50932255, gi|51535545). Asterisks on the top of the alignment indicate conserved Cystein residues (red: present in almost all DCD domains, green: present subfamily-specific)

**Figure 2:**
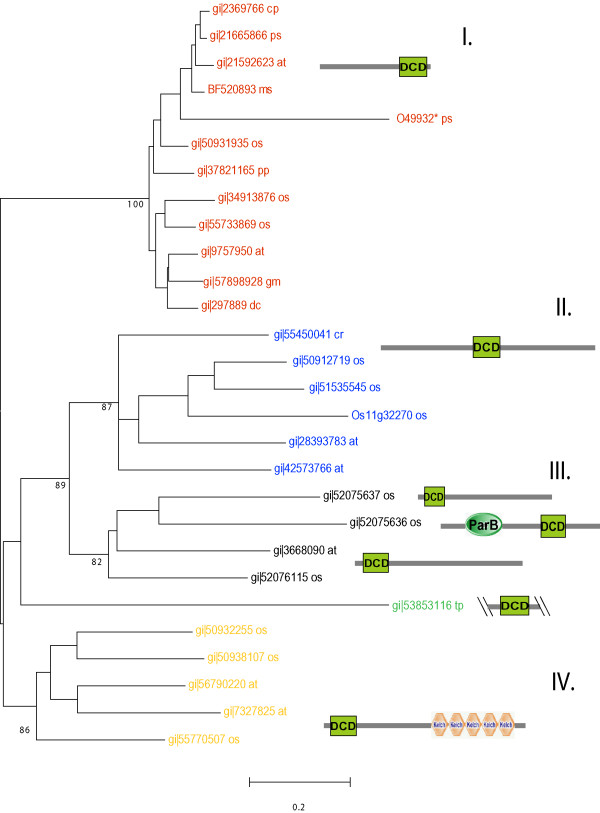
**Phylogenetic tree of DCD domains in plants and related domain architecture. **The tree topology was calculated using the Neighbor-Joining algorithm. Hypothetical proteins putatively involved in cell death with c-terminal DCD domains (I. Genbank accession numbers in red), hypothetical proteins with central DCD domains (II. Genbank accession numbers in blue), further hypothetical proteins with N-terminal DCD-domains, ParB domain preceding a DCD-domain and of unclear architecture (III. Genbank accession numbers in black), Kelch-repeat containing proteins (IV. Genbank accession numbers in yellow). The not clearly grouped sequence of *Thalassiosira *is colored in green. The accession numbers are followed by species names (at: *Arabidopsis thaliana*; cp: *Citrus X paradisi*; cr: *Ceratopteris richardii*; dc: *Daucus carota*; gm: *Glycine max*; mt: *Medicago truncatula*; os: *Oryza sativa*; pp: *Physcomitrella patens*; ps: *Pisum sativum*; tp: *Thalassiosira pseudonana*) Numbers in nodes indicate bootstrap values (only essentials are shown). The domain names are according to the Simple Modular Architecture Research Tool [19, 20]  * this sequence contains may be an incorrectly sequenced C-terminal part

Sequence analysis revealed a significantly conserved region, hence novel domain DCD. The DCD domain is an approximately 130 amino acid long stretch that contains several mostly invariable motifs (Fig. 1). These include a FGLP and a LFL motif at the N-terminus and a PAQV and a PLxE motif towards the C-terminus of the domain. Several amino acids are positionally conserved in all members with a DCD domain indicating a critical role of these residues in structure and function (Fig. 1). In particular three cysteines are almost generally (red asterisks in Fig 1) or subfamily specifically (green asterisks in Fig. 1) conserved, which putatively possess a metal binding feature. The predicted secondary structure is mostly composed of beta strands and confined by an alpha-helix at the N- and at the C-terminus. Using the metaserver 3D-Jury [7] no similarities to any other known structural folds could be assigned. The modular nature of the DCD domain is supported by the presence in several protein families with different domain architecture (Fig. 2). The DCD domain is only found in plant proteins but absent from bacteria, fungi and animals. The two fully sequenced plant genomes from rice and *Arabidopsis *contain 11 and 7 members with a DCD domain, respectively. At least four subgroups of proteins can be identified by phylogenetic comparison of the DCD domain each having members in the rice and in the *Arabidopsis *genome (Fig. 2). A similar picture emerges from the analysis of plant EST-sequences, which also cluster to the different subgroups (data not shown). The four subgroups differ in the architecture where the DCD domain is located within the protein (Fig. 2). Whereas in subgroup I the DCD domain is found in the C-terminus of the protein, it is found more towards the middle of the protein in subgroup II. The third (III) subgroup is more variable; the proteins are mostly characterized by a DCD domain at the N-terminus and in one case it is found subsequent to a ParB domain. The fourth (IV) subgroup shares a DCD domain at the N-terminus but contains several KELCH repeats at the C-terminal part of the protein.

Whereas the majority of DCD domains (families II, III. IV) contain a second conserved cysteine, directly following the N-terminal one, family I possess a putative functional substitute in the "central loop" of the domain (Fig. 1, green asterisks on the top of the alignment).

We could only identify the DCD domain in a variety of plants, using PSI-BLAST. The domain seems to be present in ESTs from dicots (e.g. *Arabidopsis*), monocots (e.g. rice), gymnosperm trees (e.g. pine), ferns, and mosses (e.g. *Physcomitrella*). The available sequences from algae are very limited, but the recently sequenced diatom *Thalassiosira pseudonana *[8] contains a distant member of this domain in a hypothetical protein (Fig. 2). At least the DCD domain is present from early in plant evolution before the separation of diatoms and green algae, leading to higher plants, occurred about 1 billion years ago.

For three of the proteins with a DCD domain, all clustering into group I, some biological data have been published. These proteins include the B2-protein from carrot, which was found to be strongly and early induced during the developmental shift from undifferentiated cell cultures to somatic embryogenesis [9]. Though the exact function of the protein still has to be elucidated a role in developmental processes is supported by the finding from *Arabidopsis *transcript profiling with microarrays. Here the DCD containing protein At2g32910 is only weakly expressed throughout the whole life cycle of *Arabidopsis *except during embryogenic development. A similar pattern is observed for the gene At5g01660, which has several KELCH repeats next to the DCD domain. This gene is most abundantly expressed in embryos but also in the meristem of the shoot apex.

A second protein with a DCD domain was identified by [10] in pea. Here the so called *Gda1 *gene is strongly expressed in peas during the vegetative phase but rapidly disappears after shifting the plants into the reproductive phase. The transition is mediated by a change of the light period from short to long days. Interestingly the *GDA1 *gene can be rapidly induced by the phytohormone gibberellic acid, a key player in the developmental change from the vegetative to the reproductive phase in plants. The *GDA1 *transcript accumulated only 15 min after application of gibberellic acid, indicating that the *GDA1 *gene is a primary response gene to this phytohormone.

A third protein with a DCD domain was isolated by [6]. This protein was named N-rich protein (NRP) because of the extreme high content of asparagine (~25%) in the N-terminal half in front of the DCD domain. The NRP-gene is rapidly induced during programmed cell death in soybean, caused by inoculation with avirulent bacteria. Isogenic bacteria, lacking a single *Avr*-gene are not recognized by soybean cells and neither trigger programmed cell death nor the induction of the *NRP *gene. The gene is induced early in the cell death program well before the cells lose control of their membrane integrity. Using *Phytophthora *as a fungal pathogen to inoculate soybean plants, the same response was found as with bacteria, indicating that the *NRP*-gene is responding to the cell death program rather than to specific molecules from a particular pathogen. The putative *Arabidopsis *ortholog (At5g42050) is induced by several stress conditions including ozone, osmotic and cold stress as indicated by publicly available transcript profiling data (Genevestigator: ). Ozone treatment leads to small lesions with cell death similar to a hypersensitive reaction caused by avirulent pathogens. A similar set of genes is activated by both inducers of programmed cell death.

The DCD domain is quite well conserved on the amino acid level throughout the plant kingdom. The domain is present in proteins with different architectures. Some of these proteins contain additional recognizable motifs, like the KELCH repeats or the ParB domain. The latter domain has been attributed to the partitioning of plasmids and chromosomes in bacteria and has a nuclease activity [11].

KELCH motifs are typically composed of ~50 amino acid long stretches which form a beta sheet [12]. They occur as 5 to 7 repeats that form a beta propeller tertiary structure. KELCH motifs are widespread and have been identified in viruses, plants, fungi and mammals. Most of the characterized KELCH motifs are interfaces for protein protein interaction, often by interaction with proteins from the cytoskeleton [13].

## Conclusion

The occurrence of the conserved DCD domain in plant proteins of variable length and different architecture, but present throughout the plant kingdom, suggests a role in protein-protein interaction. Transcription profiling reveals that the genes encoding a DCD domain are upregulated during plant development and programmed cell death. It is tempting to speculate, that the DCD domain mediates the signaling in these processes and could thus be used to identify interacting proteins to gain further molecular insights into these processes.

## Methods

Using the protein sequence of *Glycine max *(gi|57898928) as query for a PSI-Blast search [14] after one iteration we retrieve homologs in several plant families with high significance (E-value > = 8*e^-30^). A conserved region of ~130 amino acids could be identified and the borders of the shared region were defined according to the PSI-BLAST pairwise alignments. Further PSI-BLAST searches with this region converge within the first iteration. A multiple sequence alignment was build using T-coffee and refined manually; additional HMM searches [15] with profiles based on this alignment of non redundant representatives support the findings.

Phylogenetically distant sequences of diatom (*Thalassiosira pseudonana*), fern (*Ceratopteris richardii*) and moss (*Physcomitrella patens*) derive from searches against genome database and EST database, respectively.

Two different methods, Phd/PROF_sec [16] and Psipred [17], were used to predict the secondary structure.

A phylogenetic tree was reconstructed using the non redundant alignment of 29 sequences (including fragments and one translation (O49932) that likely contains a frameshift at the C-terminus) in MEGA [18], calculated with the neighbor-joining algorithm. Similar topologies were obtained using other methods e.g minimum evolution (data not shown) and bootstrap values were calculated to test significance. The domain architecture is predicted and displayed by the Simple Modular Architecture Research Tool [19,20].

## Authors' contributions

RT carried out the molecular genetic studies and initially identified the described domain.

TD performed the sequence based and phylogenetic analysis and PB coordinates the project. All authors contributed to the writing of the manuscript.
